# Electrical properties, substrate specificity and optogenetic potential of the engineered light-driven sodium pump eKR2

**DOI:** 10.1038/s41598-018-27690-w

**Published:** 2018-06-18

**Authors:** Christiane Grimm, Arita Silapetere, Arend Vogt, Yinth Andrea Bernal Sierra, Peter Hegemann

**Affiliations:** Experimental Biophysics, Institute for Biology, Humboldt-Universität zu Berlin, Invalidenstr. 42, 10115 Berlin, Germany

## Abstract

A new microbial rhodopsin class that actively transports sodium out of the cell upon illumination was described in 2013. However, poor membrane targeting of the first-identified sodium pump KR2 in mammalian cells has hindered the direct electrical investigation of its transport mechanism and optogenetic application to date. Accordingly, we designed enhanced KR2 (eKR2), which exhibits improved membrane targeting and higher photocurrents in mammalian cells to facilitate molecular characterization and future optogenetic applications. Our selectivity measurements revealed that stationary photocurrents are primarily carried by sodium, whereas protons only play a minor role, if any. Combining laser-induced photocurrent and absorption measurements, we found that spectral changes were not necessarily related to changes in transport activity. Finally, we showed that eKR2 can be expressed in cultured hippocampal mouse neurons and induce reversible inhibition of action potential firing with millisecond precision upon illumination with moderate green-light. Hence, the light-driven sodium pump eKR2 is a reliable inhibitory optogenetic tool applicable to situations in which the proton and chloride gradients should not be altered.

## Introduction

Microbial rhodopsins are photosensitive retinylidene proteins that are utilized by fungi, algae, and prokaryotes to sense and adapt to light or to harness light energy^1,2^. Microbial rhodopsins contain a seven transmembrane helical structure enclosing an all-*trans* retinal^3–5^ chromophore that is covalently bound to helix seven via a Schiff base linkage. A subset of microbial rhodopsins functions as ion pumps that use light energy to transfer specific ions across the membrane by vectorial transport. Over the last decade, neuroscientists have used these light-activated transporters including Arch3, an outward-directed H^+^ pump, and *Np*HR, an inward-directed chloride pump, to hyperpolarize excitable cells and inhibit action potential (AP) firing in neurons with unprecedented spatiotemporal precision^6–8^.

In 2013, Inoue *et al*.^9^ reported the existence of a light-driven Na^+^ pump (KR2) in the flavobacterium *Krokinobacter eikastus*. In the following years, several homologous members of this new class of microbial pumps were identified in other marine^10–12^ and freshwater bacteria^13,14^. Due to its high expression levels in *E*.*coli*, recombinant KR2 was purified in large amounts, which enabled numerous *in*-*vitro* studies and crystallization within two years of its discovery^15,16^. When activated by light, KR2 pumps Na^+^ out of the cell, as determined from pH measurements in suspensions of KR2-expressing *E*. *coli* cells^17^. However, based on this assay, it was also suggested that KR2 actively transports H^+^ in the absence of extracellular Na^+^ ^9^. *In*-*vitro* measurements of KR2 reconstituted into black lipid membranes (BLMs) support the assumption of an active, light-induced Na^+^ transport by KR2 and show that transport activity prevails when Na^+^ is removed, which could be explained by alternative H^+^ pumping^9,16^.

Although KR2 has been studied extensively over the past five years, electrophysiological measurements that directly monitor the activity of the pump are limited. In particular, voltage-clamp experiments in mammalian cells aimed at investigating light-induced ion transport under defined intra- and extracellular ionic conditions and under the control of the membrane voltage are still lacking. In contrast to spectroscopic measurements of KR2 in detergent or native lipid nanodiscs, electrical whole-cell recordings allow asymmetric variations with different ionic conditions on the intra- and extracellular side. However, because membrane targeting of wild-type (wt) KR2 in mammalian cells is poor^18,19^, direct electrophysiological investigations of the transport properties and the substrate specificity of Na^+^ pumps have been hindered.

To enable electrical measurements of KR2, we modified the N- and C-termini, which improved expression in mammalian ND7/23 cells. One of the variants generated, denoted enhanced KR2 (eKR2), showed excellent membrane targeting and 60-fold greater photocurrents than the unmodified, wt protein and photocurrents that were six times higher than the C-terminally modified KR2 variant used in prior studies^15,18^. Using eKR2, we investigated photocurrent dependence on intra- and extracellular [Na^+^] or [H^+^] to elucidate the proposed dual pumping of Na^+^ and H^+^ by KR2^9,14^, and found that eKR2 is a light-driven Na^+^-selective ion pump with minimal K^+^ pump activity. However, we did not observe a significant contribution of H^+^ transport to the stationary photocurrent, even at low intracellular [Na^+^]_i_. We correlated spectroscopic flash-photolysis data recorded at different [Na^+^] and pH values with laser-induced single turnover measurements of eKR2 photocurrents generated in mammalian cells under similar buffer conditions. Further, we demonstrated that the enhanced expression of eKR2 is preserved in cultured hippocampal mouse neurons and enabled the inhibition of AP firing upon moderate green-light illumination, rendering eKR2 a potent optogenetic tool for efficiently silencing neurons. In contrast to other inhibitory tools, Na^+^-based silencing with eKR2 would neither influence the distribution of H^+^ or Cl^−^ nor rely on the anion gradient.

## Results

### Molecular design and spectral characterization of eKR2

**Figure 1:**
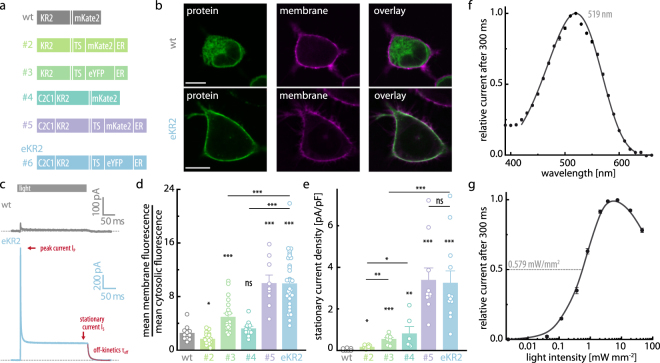
Molecular design of eKR2 and its spectral characteristics. (**a**) KR2 variants with different modifications at the N- or/and C- terminus to improve membrane targeting. (**b**) Representative confocal images (0.5-µm equatorial slices) of ND7/23 cells expressing the wild type (top) and the best construct eKR2 (bottom) showing the fluorescence of the fusion protein in green (left), the membrane marker in magenta (middle), and their co-localization in white (false colors); scale bar 10 µm. (**c**) Representative photocurrents of wild-type KR2 (grey) and eKR2 (light blue) recorded from ND7/23 cells (525 nm, 45 mW mm^−2^). (**d**) Membrane targeting of all constructs determined from the confocal images (mean ± SEM with individual data points); *p < 0.05, **p < 0.01, ***p < 0.001 compared to wild type or the indicated variant. (**e**) Light-induced (525 nm) stationary currents at 0 mV recorded from ND7/23 cells (symmetric 110 mM [Na^+^], pH 7.2) for all variants (mean ± SEM with individual data points); *p < 0.05, **p < 0.01, ***p < 0.001 compared to wild type or the indicated variant. (**f**) Normalized photocurrent amplitude (0 mV) of eKR2 in ND7/23 cells at different wavelengths (equal photon flux) of activation light (mean ± SEM; n = 6); fitted with a Weibull function, symmetric 110 mM [Na^+^], pH 7.2. (**g**) Stationary photocurrent (mean ± SEM; n = 6) of eKR2 in ND7/23 cells upon increasing intensities of activation light (525 nm); normalized to maximal value, symmetric 110 mM [Na^+^]_i/e_, pH 7.2.

To elucidate the KR2 pumping mechanism and its substrate specificity, we attempted an in-depth electrophysiological characterization of KR2 in mammalian cells, which controls the intra- and extracellular ionic composition as well as the membrane voltage. We found that wt KR2 exhibits negligible light-induced currents in neuronal ND7/23 cells (Fig. 1c). Thus, we expressed several KR2 variants containing N- or/and C-terminal modifications (Fig. 1a), evaluated their membrane targeting using confocal imaging (Figs 1b,d and S1), and quantified their light-induced activity using patch-clamp recordings (Fig. 1e). A combination of C-terminal targeting sequences (TS and ER^20,21^) and the N-terminal fusion of KR2 to a channelrhodopsin N-terminus^22^ yielded a variant (#5) with excellent membrane targeting and light-induced currents 65-fold higher than the unmodified protein (Fig. 1d,e). We exchanged the mKate2 fluorophore with eYFP (#6), because eYFP is a commonly used fluorescence tag in neuroscience, which yielded improvements from construct #2 to #3, but had no influence at this stage of the exchange. Construct #6 exhibited nearly exclusive membrane targeting (Fig. 1b,d) combined with photocurrents that were 60-fold higher than the wt KR2 (Fig. 1c,e); thus, we called the construct eKR2 and used it to conduct our electrical characterization. We also tested eKR2 in *Xenopus laevis* oocytes, in which it also produced significantly higher photocurrents than wt KR2 (p < 0.001, Supplemental Fig. S2).

To identify the best activation wavelength for eKR2, we recorded an action spectrum of the stationary photocurrent (*I*_s_) using symmetric 110 mM [Na^+^]_i/e_ (pH_i/e_ 7.2), and determined the maximal *I*_s_ occurred at 519 nm (Fig. 1f), which was in agreement with the maximal dark state absorption of the purified protein (Supplemental Fig. S3). Additionally, we titrated *I*_s_ with the intensity of the actinic light (525 nm) and found that the half-maximal amplitude was reached at 0.579 mW/mm^2^. Interestingly, the photocurrents decreased again after reaching a maximal value at approximately 7 mW/mm^2^, reducing the *I*_s_ to around 80% at full light intensity (46 mW/mm^2^). This result indicated a secondary photochemical reaction occurred at high light intensities that diminished the effective *I*_s_ via the photoactivation of at least one of the photocycle intermediates.

### eKR2 photocurrents at different intracellular ionic conditions

Using eKR2, we investigated the substrate specificity of the pump in mammalian ND7/23 cells at defined intracellular ionic conditions representing the substrate pool for an outward-directed pump. Because we noted that eKR2 pumps K^+^ to a certain extent (Supplemental Fig. S4) in preliminary experiments, we replaced Na^+^ with the non-transported N-methyl-D-glucamine (NMG^+^) whenever we lowered [Na^+^].

**Figure 2:**
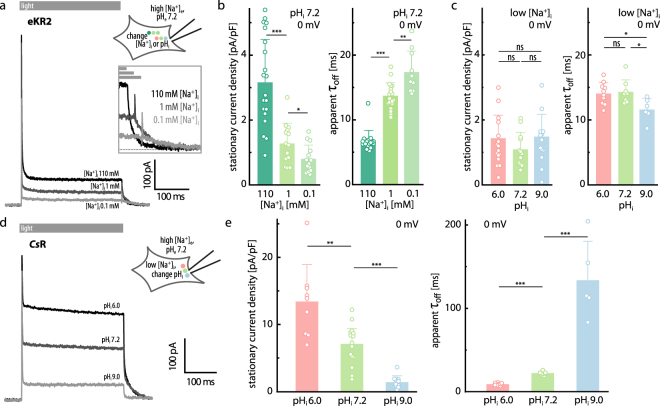
Photocurrents of eKR2 and *Cs*R in ND7/23 cells at 0 mV and varying intracellular ionic compositions. (**a**) Representative photocurrents of eKR2 after illumination with 525 nm light (0 mV) at different intracellular [Na^+^]_i_ at 110 mM [Na^+^]_e_ and pH_e_ 7.2 with the off-kinetics (inset). (**b**) Stationary photocurrents of eKR2 normalized to respective cell capacitance extracted from measurements at different intracellular [Na^+^]_i_ (pH_i_ 7.2) and corresponding apparent time constants from the bi-exponential decline of these currents after light-off; 110 mM [Na^+^]_e_, pH_e_ 7.2 (mean ± SD and individual data points, 0 mV). (**c**) Stationary photocurrents of eKR2 normalized to cell capacitance extracted from measurements at different intracellular pH values ([Na^+^]_i_ 1 mM) and corresponding apparent time constants from the bi-exponential decline of the currents after light-off; 110 mM [Na^+^]_e_, pH_e_ 7.2 (mean ± SD and individual data points, 0 mV). (**d**) Representative photocurrents of the H^+^ pump *Cs*R after illumination (bar) with 525 nm light at 0 mV and intracellular pH_i_ 6.0, 7.2, and 9.0; 1 mM [Na^+^]_i_ (110 mM [Na^+^]_e_, pH_e_ 7.2). (**e**) Stationary photocurrents of *Cs*R normalized to cell capacitance extracted from measurements at conditions described in (**d**) and corresponding apparent time constants from the bi-exponential decline of the currents after light-off (mean ± SD and individual data points, 0 mV).

To probe the reaction of a pump to varying substrate concentrations, we measured the photocurrents of *Cs*R, the H^+^ pump of *Coccomyxa subellipsoidea*^23,24^, at various intracellular pH_i_ values and constant external [Na^+^]_e_ (110 mM, pH_e_ 7.2) (Fig. 2d). The *Cs*R photocurrent size (0 mV holding voltage) depended on the substrate concentration and decreased when the intracellular [H^+^]_i_ was lowered from pH_i_ 6.0 to pH_i_ 7.2 and pH_i_ 9.0, which is in accord with previously published data for H^+^ ^25^ and Cl^−^ pumps^6,26^. Additionally, the decreasing *I*_s_ was accompanied by slowed kinetics of the biphasic current decay (*τ*_off_) after the light was switched off (Fig. 2e).

In a similar experiment with eKR2-expressing ND7/23 cells, the *I*_s_ remained unaffected by variation in the intracellular [H^+^]_i_ from pH_i_ 6.0 to pH_i_ 7.2 and pH_i_ 9.0 at 1 mM [Na]_i_ (110 mM [Na^+^]_e_, pH_e_ 7.2; Fig. 2c). Additionally, the eKR2 *τ*_off_ values did not systematically depend on the intracellular [H^+^]_i_, and the photocurrent even decayed faster at pH_i_ 9.0 (Fig. 2c), which is inconsistent with H^+^ pumping. In contrast, variation in [Na]_i_ from 110 mM to 1 mM and 0.1 mM (Fig. 2a,b) significantly decreased the eKR2 photocurrents and slowed the kinetics accordingly. This dependence was likewise observed at 1 mM external [Na^+^]_e_ (Supplemental Fig. S5), indicating that amplitude and kinetics were defined by the substrate availability and not by the Na^+^ gradient. Figure 2a depicts typical photocurrent traces at different intracellular [Na^+^]_i_ (pH_i_ = 7.2), featuring a high peak current (*I*_p_) that decayed bi-exponentially (*τ*_app,inac_ = 2.0 ± 0.3 ms at 110 mM) to a lower *I*_s_ at constant illumination, which decayed bi-exponentially back to baseline after light termination. Interestingly, at low internal [Na^+^]_i_ (0.1 mM or 1 mM), the currents displayed a small overshot upon light-off (Fig. 2a, inset). Similarly, the inactivation kinetics from *I*_p_ to *I*_s_ were also dependent on the intracellular [Na^+^]_i_ (Supplemental Fig. S6).

### Influence of the external ion composition and dependence on membrane voltage

**Figure 3:**
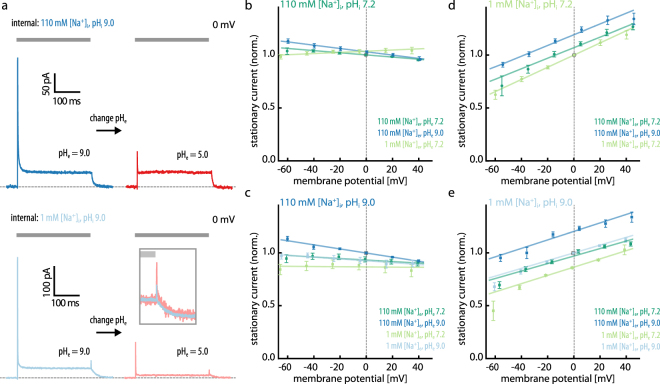
Voltage dependence of eKR2 photocurrents at different intra- and extracellular ionic compositions. (**a**) Photocurrent traces of eKR2 at high H^+^ gradients (0 mV). Photocurrents from the same cell at symmetric pH_i_ 9.0 (left) and after the extracellular buffer was exchanged to pH_e_ 5.0 (right) to create a high H^+^ gradient; top: 110 mM symmetric [Na^+^]_i/e_; bottom: 1 mM symmetric [Na^+^]_i/e_. (**b**) Voltage dependence of the stationary photocurrents of eKR2 at 110 mM [Na^+^]_i_, pH_i_ 7.2 and different extracellular pH_e_ values and [Na^**+**^]_e_ as indicated (normalized to the symmetric condition; LJP corrected; mean ± SEM, n = 6, 5, and 6). (**c**) Same experiment as in (b) but at 1 mM [Na^+^]_i_, pH_i_ 7.2 (normalized to the symmetric condition; LJP corrected; mean ± SEM, n = 8, 11, 8, and 6). (**d**) Same experiment as in (b) but at 110 mM [Na^+^]_i_, pH_i_ 9.0 (normalized to the symmetric condition; LJP corrected; mean ± SEM, n = 7, 7, and 7). (**e**) Same experiment as in (b) but at 1 mM [Na^+^]_i_, pH_i_ 9.0 (normalized to the symmetric condition; LJP corrected; mean ± SEM, n = 6, 5, 6, and 6).

Despite a lack of evidence so far, we further explored the capability of eKR2 to transport H^+^. Several decades ago, studies demonstrated that high H^+^ gradients against the pumping direction inhibit the H^+^ pump bacteriorhodopsin, a phenomenon called the back pressure effect^27,28^. Thus, we established the whole-cell configuration at symmetric pH 9.0_i/e_ and different intracellular [Na^+^]_i_ (Fig. 3a, left), and exchanged the extracellular buffer to pH_e_ 5.0 to create a high H^+^ gradient (load) against the pump direction (Fig. 3a, right). At symmetric 110 mM [Na]_i/e_ (Fig. 3a, top), a change in [H^+^]_e_ from pH_e_ 9.0 to pH_e_ 5.0 reduced *I*_p_ to 30% but did not affect *I*_s_, suggesting that the *I*_p_ might be carried by H^+^. Additionally reducing extracellular [Na]_e_ at external pH_e_ 5.0 left *I*_s_ unaffected (Supplemental Fig. S7), implying that the extracellular [Na^+^]_e_, along with the Na^+^ gradient, had little effect on the current. Interestingly, when the external pH was reduced at 1 mM internal [Na^+^]_i_, both the *I*_s_ and the *I*_p_ were reduced (Fig. 3a, bottom), suggesting that pumping activity was inhibited at this condition. However, in contrast to the previous experiments conducted at different intracellular [Na^+^]_i_ (Fig. 2b), the apparent reduction in *I*_s_ was not accompanied by slowed off-kinetics (Fig. 3a inset). Accordingly, we concluded that at external pH 5.0, *I*_p_ is reduced at high and low internal [Na^+^]_i_, whereas *I*_s_ is only affected when the internal [Na^+^]_i_ is low. In oocyte measurements, we observed a similar reduction in *I*_s_ at external pH_e_ 5.0 (Supplemental Fig. S2), but here the internal pH_i_ was approximately 7.5, resulting in a much smaller pH gradient. Consequently, we surmised that the photocurrent reduction was evoked by extracellular acidification (at low [Na^+^]_i_) rather than by the high H^+^ gradient, which was confirmed in ND7/23 cells as well (Supplemental Fig. S7).

Next, we investigated the influence of the applied membrane potential on the eKR2 photocurrent. We recorded the current-voltage relationship at different extracellular [Na^+^]_e_ (110 mM or 1 mM) and [H^+^]_e_ (pH_e_ 7.2 or 9.0) under select intracellular conditions to systematically investigate the influence of Na^+^ and H^+^ gradients. In summary, we found that the eKR2 *I*_s_ (i) was independent of the membrane voltage when intracellular Na^+^ was abundant (Fig. 3b and c), (ii) became voltage dependent when intracellular [Na^+^]_i_ was low (Fig. 3d and e), (iii) was slightly promoted by external [Na^+^]_e_ (110 mM) and alkaline pH_e_ 9.0 regardless of the ionic/pH gradient, and (iv) collapsed at low external pH_e_ 5.0 but only when intracellular [Na^+^]_i_ was low.

### Single turnover experiments

**Figure 4:**
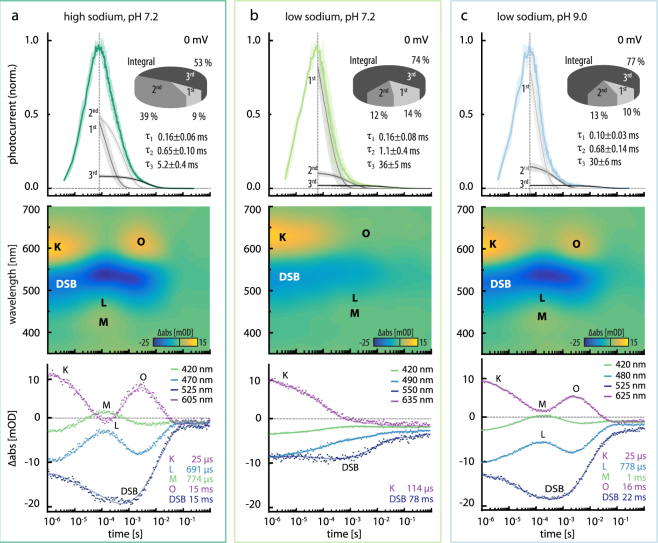
Laser-induced eKR2 photocurrents and flash photolysis with recombinant KR2. Comparison of results from different ionic and pH conditions (**a**) 110 mM [Na^+^]_i/e_ at pH 7.2 (mean ± SD, n = 3), (**b**) 1 mM [Na^+^]_i/e_ at pH_i/e_ 7.2 (mean ± SD, n = 4), and (**c**) 1 mM [Na^+^]_i/e_ at pH 9.0 (mean ± SD, n = 5). Top: Averaged normalized single turnover eKR2 photocurrents (0 mV) induced by a 7-ns laser flash (525 nm) and corresponding components of the fit of the current decay with a tri-exponential function; inset: pie chart with integrals of the three exponential decay components. Middle: contour plots from flash photolysis measurements of recombinant KR2 under the same conditions together with time traces at the indicated wavelengths. Bottom: time constants resulting from exponential fitting (decay) of the indicated time trace; points represent SVD filtered raw data, line is only guidance to the eye.

To resolve fast kinetics in the photocurrents and improve comparability to transient absorption spectral data, we measured eKR2 photocurrents in single turnover experiments (Fig. 4). We recorded the photocurrents in response to a 7-ns laser flash at three different symmetric conditions, 110 mM [Na^+^]_i/e_ at pH_i/e_ 7.2 (Fig. 4a), 1 mM [Na^+^]_i/e_ at pH_i/e_ 7.2 (Fig. 4b), and 1 mM [Na^+^]_i/e_ at pH_i/e_ 9.0 (Fig. 4c). At all of the ionic conditions tested, the laser-induced eKR2 photocurrents rose to maximal amplitudes within 60–70 µs after the laser flash (0 mV), which was beyond the limits of our time resolution in ND7/23 cells. Thus, only the photocurrent decay was fitted by a tri-exponential function, for which we plotted the resultant three components onto the recorded photocurrents (Fig. 4, top). The same Na^+^ concentrations and pH values were used to measure the transient absorption difference spectra of recombinant KR2 using flash-photolysis. The resulting spectra are represented by reconstructed surface plots (Fig. 4, middle) and time traces at the indicated wavelengths for the photointermediates and the dark state bleach (DSB) (Fig. 4, bottom). Time constants were obtained by fitting the respective time trace using an exponential function (Fig. 4, bottom).

At 110 mM [Na^+^] and pH 7.2 (Fig. 4a), the spectroscopic data revealed a fast K state decaying with *τ*_K_ = 25 µs, preceded by the L/M equilibrium, which decayed with *τ*_L_ = 691 µs and *τ*_M_ = 774 µs, and a late O state decaying with τ_O_ = 15 ms (Fig. 4a, middle/bottom and Supplemental Fig. S3), which was in accord with previously published spectroscopic data at high [Na^+^] at neutral pH^9,29^. The two fast and the slowest electrical components decayed with *τ*_1_ = 0.16 ± 0.06 ms, *τ*_2_ = 0.65 ± 0.10 ms, and *τ*_3_ = 5.2 ± 0.4 ms under these conditions, respectively (Fig. 4a, top). Although the slowest component contributed only 6 ± 1% to the photocurrent amplitude under single turnover conditions (Fig. 4 top, pie charts), it was the major contributor to the overall transported charge (53 ± 1%) based on the integrals of the fitted components. In addition, from the ms-time regime onwards, the photocurrent was solely carried by the third component, leading us to conclude that it was the dominant charge carrier of *I*_s_ recorded during continuous illumination. When [Na^+^]_i/e_ was reduced to 1 mM at pH_i/e_ 7.2 (Fig. 4b), the fastest electrical component was unchanged regarding both the relative integral (9 ± 2% *vs*. 14 ± 4%; p = 0.105, unpaired t-test) and the time constant (0.16 ± 0.06 ms *vs*. 0.16 ± 0.08 ms; p = 0.934, unpaired t-test). However, the relative amplitude and integral of the second fastest component decreased from 40 ± 1% to 7 ± 2% (p < 0.0001, unpaired t-test) and 39 ± 2% to 12 ± 2% (p < 0.0001, unpaired t-test), respectively, with a reduction in [Na^+^], but no significant changes in the respective *τ*_2_ value were observed (p = 0.081, unpaired t- test). Interestingly, we noted a significant reduction (p < 0.0001, unpaired t-test) from 5.2 ± 0.4 ms to 36 ± 5 ms in the kinetics of the slowest component *τ*_3_, which was consequently considered the rate limiting step. The spectroscopic data revealed an equilibrium between the L/M and O states (Supplemental Fig. S3) and their corresponding absorption differences were barely visible in the time traces (Fig. 4b, bottom); thus, we omitted fitting of the kinetics here. Moreover, we found that the K state decay and the recovery of the DSB could no longer be fitted with a single exponential function and the apparent decay time constants slowed with *τ*_K_ = 114 µs and *τ*_rec_,_DSB_ = 78 ms.

When both [Na^+^] and [H^+^] were reduced (1 mM [Na^+^]_i/e_, pH_i/e_ 9.0), the laser-induced photocurrents (Fig. 4c) did not change qualitatively compared to low [Na^+^]_i/e_ at pH_i/e_ 7.2 (Fig. 4c, top). However, the O state (τ_O_ = 16 ms) was no longer in equilibrium with the L/M states in flash photolysis experiments (Fig. 4c), and the overall photocycle kinetics were faster than the corresponding values at low [Na^+^] pH 7.2, with *τ*_K_ = 25 µs, *τ*_L_ = 778 µs, *τ*_M_ = 1 ms, and *τ*_rec,DSB_ = 22 ms. Although the distinct O state absorption difference reappeared, it was less pronounced than at high [Na^+^] and pH 7.2. The cause of this effect appeared to be a change in pH independent of the [Na^+^]. Furthermore, at high [Na^+^] (150 mM), we observed that the O state was more pronounced in alkaline conditions (Supplemental Fig. S8).

### Silencing of mouse hippocampal neurons in culture

Previous attempts to silence neuronal activity using light-induced sodium pumping involved a construct similar to our construct #3^15,19^, which exhibited poor membrane targeting and negligible photocurrents in our ND7/23 cell measurements. However, the eKR2 construct showed improved membrane targeting and higher photocurrent amplitudes in ND7/23 cells compared to construct #3 (Fig. 1c and d). Thus, we decided to test if the observed improvements translated to enhanced performance of eKR2 in silencing neuronal activity in primary cultures of mouse hippocampal neurons.

**Figure 5:**
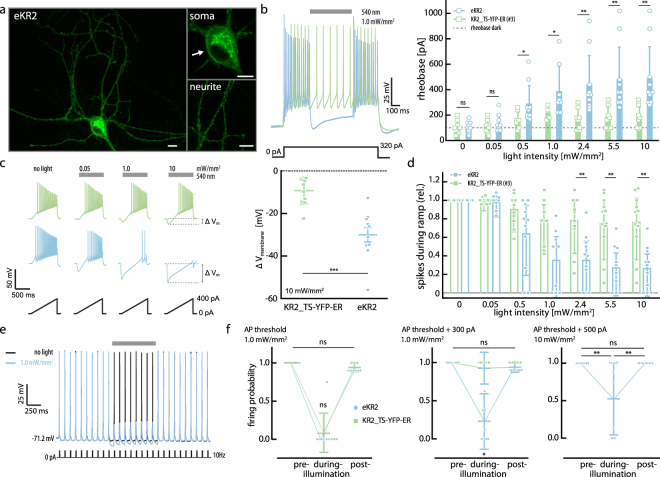
Neuronal silencing performance of eKR2. (**a**) Confocal image of a hippocampal neuron in culture (Z stack) with zoom-ins (0.5-µm equatorial slice) on dendrites and soma; scale bar corresponds to 10 µm. (**b**) Left: Representative voltage traces recorded in current-clamp configuration using a squared somatic current injection protocol to determine the rheobase (540 nm LED, 500 ms) Right: Dependence of average rheobase on illumination intensity (mean ± SD; n = 10). Dotted line represents the average rheobase without illumination. (**c**) Left: current-clamp traces at different light intensities using a somatic current injection ramp protocol with 0.44 pA/ms (0 to 400 pA in 900 ms). Right: Cell hyperpolarization at 10 mW/mm^2^ green light stimulation (mean ± SD; n = 11). Delta membrane voltage was calculated as the minimum membrane voltage value under light stimulation minus the membrane voltage at 0 somatic current injection and no light stimulus. (**d**) Relative number of spikes during the ramp protocol at different light intensities normalized to no light stimulation (mean ± SD; n = 11). (**e**) Pulse trial protocol traces of an eKR2-expressing neuron with (light blue) and without (black) light stimulation; pulse width 10 ms, LJP corrected. (**f**) Firing probability during pulse trial protocol pre-, during, and post- light stimulation (mean ± SD; n = 11). Left: At AP threshold; middle: injection amplitudes at AP threshold plus 300 pA. Right: At AP threshold plus 500 pA (only eKR2). The Kruskal Wallis test was used followed by Dunn’s test.

Confocal imaging revealed good membrane targeting of eKR2 in mouse hippocampal neuron cultures (Fig. 5a). Although construct #3 contained eYFP flanked by the endoplasmic reticulum (ER) and the Golgi export (TS) sequences, it still resulted in the previously observed accumulation of protein in soma and neurites (data not shown)^15^. However, the eKR2 construct overcame these problems. To test the ability of our constructs to inhibit neuronal activity, we measured the minimum amount of sustained current necessary to initiate an AP (rheobase) for both constructs at different light intensities (Fig. 5b). Under light stimulation - already at 0.5 mW/mm^2^ - eKR2 had a significantly stronger impact on the rheobase compared to construct #3, and this effect was further increased at higher light intensities. At 2.4 mW/mm^2^, eKR2 produced a rheobase shift two times higher than construct #3 (Fig. 5b). We also applied a somatic ramp current injection protocol to test the effect of our constructs on neuronal excitability (Fig. 5c). Notably, construct #3 only decreased the average number of AP fired by a neuron to 76% at high light intensities (Fig. 5d) and never managed to completely abolish AP firing during the ramp protocol. Conversely, the average number of AP fired during the ramp following eKR2 activation was reduced to 26%, and in some cases completely abolished, which is explained by the higher light-induced hyperpolarization produced by eKR2 compared to construct #3 (Fig. 5c, right). An important aspect for application as an optogenetic tool is reversibility of light-induced effects. Consequently, we used a pulse trial protocol to test if the neurons returned to normal neuronal activity after light stimulation (Fig. 5e). Both constructs decreased the probability of AP firing when the light was applied and the pre-illumination AP firing pattern was restored when the light was switched off. Nonetheless, eKR2 still reliably inhibited AP firing at a higher current injection (Fig. 5f).

## Discussion

The discovery of light-driven Na^+^ pumps expanded the family of microbial rhodopsins by inclusion of a new member with unprecedented properties. Although the excellent expression of wt KR2 in *E*. *coli* enabled spectroscopy and X-ray crystallography studies, the poor membrane targeting in mammalian cells hampered direct electrical characterization, which is indispensable for understanding the pumping mechanism and evaluating the applicability of KR2 for optogenetic applications. Our eKR2 variant overcame these former limitations and enabled direct electrophysiological studies of the pumping activity under different (and asymmetric) ionic conditions or membrane voltages.

In the case of eKR2, *I*_s_ increased gradually with intracellular [Na^+^]_i_ (pH_i_ 7.2) due to the acceleration of the photocycle, confirming the Na^+^ pumping activity of eKR2 in accord with the original hypothesis^9^. However, in contrast to previous claims, we did not generate evidence to support the contribution of H^+^ to *I*_s_. Intracellular H^+^ did not promote *I*_s_ or accelerate the photocycle kinetics. Conversely, a change in pH_i_ from 9.0 to 7.5 (1 mM [Na^+^]_i_) slowed the kinetics (Fig. 2), which disputes the H^+^ transport hypothesis. However, the possibility that the H^+^ affinity is so high to render the photocycle entirely independent of H^+^ in the observed pH range cannot be ruled out entirely.

The profound reduction in *I*_s_ at increased extracellular [H^+^]_e_ (pH_e_ 9.0 to 5.0) and low intracellular [Na^+^]_i_ might be interpreted as H^+^ pumping shut-off. However, because the kinetics were not altered, we interpret this loss of activity as protein inactivation, which might be mediated by a titratable side chain in or close to the putative extracellular ion release cavity involved in transient Na^+^ binding^16^, consequently enhancing activity at alkaline pH and impairing function at pH_e_ 5.0. In accord with our interpretation, previous studies have shown that individual elimination of the negative charges E11 and E160 reduced pumping activity^16^.

**Figure 6:**
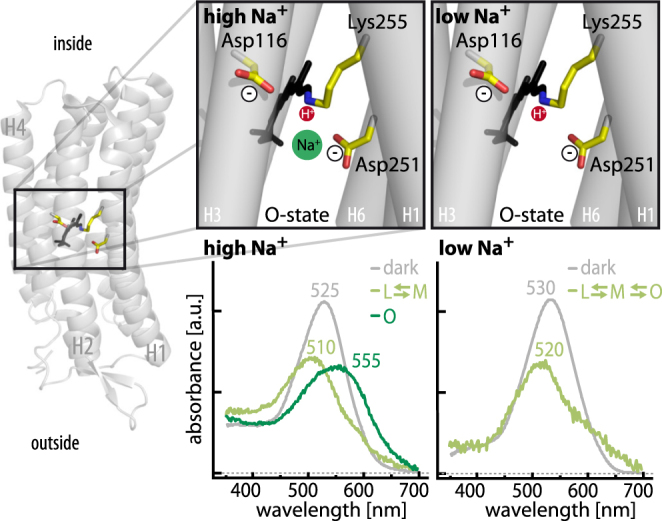
Red-shift of O-state absorption by Na^+^. Structure of KR2 (PDB:4XTL)^16^ on the left with close up on the proposed transient Na^+^ binding site close to D251^15,31,49^. At high [Na^+^] the presence of the charge (middle) caused a red-shift of the retinal absorption in the O state compared to the ground state, whereas the absorption was similar to the ground state absorbance in absence of Na^+^ (right). Scheme created using the dark state structure, no implications regarding the retinal isomerization state intended; helix2 is removed for visibility.

Fitting the decay of laser-induced photocurrents revealed two sub-ms decay components constituting the transient current amplitude and a slower component mainly carrying the current in the ms-time regime. These three electrical components agree with previous findings describing three kinetic transmembrane charge transfer steps in similar time regimes for the NaR of *Dokdonia sp*. PRO 95^30^. Assuming that one charge per photon is pumped under all conditions, the same net charge is always transported regardless of the individual transport steps, with each step representing a charge displacement within the protein. The second fastest electrical decay constant *τ*_2_ was kinetically independent of [Na^+^], but its’ relative contribution to the overall current markedly decreased following Na^+^ removal, implying the distance of charge displacement within the protein is affected by [Na^+^]. It could be assumed that *τ*_2_ correlates to the transition from the L/M to the O-state, which was previously thought to be Na^+^ dependent^9^ and exhibited similar time constants in our spectroscopic experiments. According to theoretical calculations reported by Suomivuori *et al*.^31^, this transport step would correspond to the translocation of Na^+^ past the retinal Schiff base, which is thought to occur during the L/M to the O-state transition. Moreover, the entire photocycle slowed down at low [Na^+^]_i/e_, which was reflected in the spectroscopic data and, particularly in the slow electrical component. Although the distinct O-state absorption difference reportedly prevailed to a small extent for Inoue and colleagues^9^, it was not observed (1 mM [Na^+^], pH 7.2) in the current study. However, in the prior experiment, Na^+^ was replaced by K^+^, which is also pumped, and caused a small O-state absorption difference in our high [K^+^] experiment (Supplemental Fig. S9). Symmetrically reducing [H^+^]_i/e_ at 1 mM [Na^+^]_i/e_ did not affect the electrical components, implying a similar transport mechanism under these conditions, whereas the transient absorption spectra changed substantially and the O-state absorption difference reappeared, indicating that the prominent spectroscopic changes were not necessarily linked to functional differences. This finding led us to conclude that although the dark state absorption was only slightly affected (Supplemental Fig. S3), the O-state absorption was significantly red-shifted by Na^+^ binding, which was also modulated by [H^+^] (Supplemental Fig. S8). Consequently, the transient (pH-dependent) binding of positively charged Na^+^ near the RSB^15,16,31^ red-shifts the retinal absorption away from the dark state absorption, rendering the O-state visible. Conversely, the O-state conformation without Na^+^ resembled the dark state with a similar absorption, masking it in the absorption difference measurements (Fig. 6). This finding was also supported by the reduced DSB at low [Na^+^] and pH 7.2, which implies the rise of a photointermediate with an absorption similar to the dark state.

Finally, we evaluated the silencing performance of eKR2 in cultured hippocampal neurons and showed that the protein was non-toxic, highly expressed, and that it reliably inhibited AP firing upon green light illumination. Already at a moderate light intensity of 0.5 mW/mm^2^ (540 nm LED), eKR2 produced a significant rheobase shift, and its silencing capabilities were further improved upon increasing the light intensity with an average rheobase shift of (333 ± 237) pA at 10 mW/mm^2^, our highest but still modest intensity (Fig. 5b).

Currently, there are several optogenetic actuators that efficiently silence neuronal activity as recently reviewed by Wiegert *et al*.^32^. However, to date rhodopsin-based silencing tools rely on passive Cl^−^ transport^33–35^, active Cl^−^ ^21,36^, or active H^+^ pumping^8^. Further, their main drawbacks are the dependence on the variable Cl^−^ reversal potential or adverse effects on pH^37–39^ or Cl^−^ distribution, especially in small subcellular compartments. Accordingly, a Na^+^ pump that extrudes only Na^+^ from the cell should provide a less invasive means of neuronal silencing. Recent attempts to enhance the poor performance of wt KR2 included the generation of a chimeric NaR^18^ and the search for other natural NaRs with improved performance^19^. Although previously reported variants only hyperpolarized the membrane up to 10 mV upon illumination, eKR2 activation produced a 3-fold higher mean hyperpolarization of 30 mV, which enabled reliable and reversible inhibition of neuronal AP firing upon illumination. With its enhanced silencing performance, eKR2 adds to the optogenetic toolbox and is a valuable alternative to silencing neurons with ms-precision, especially in contexts in which inhibition based on H^+^ or Cl^−^ transport is unfavorable or impossible.

## Material and Methods

All experiments were conducted in accordance with the guideline given by Landesamt für Gesundheit und Soziales Berlin and were approved by this authority.

### Molecular biology

All genes were synthesized by GenScript (Nanjing, China). For electrophysiological recordings from *Xenopus* oocytes the human codon optimized gene was cloned into the pGEM-HE vector. The mRNA was transcribed *in vitro* using the mMESSAGE mMACHINE® T7 Transcription Kit (Thermo Fisher Scientific, Waltham, MA, USA).

For patch-clamp recordings in ND7/23 cells, the human/mouse codon-optimized DNA sequence of KR2 fused to a channelrhodopsin N-terminus^22,40^ was synthesized and cloned into a pmKate2-N1 plasmid (CMV promotor, BamHI/AgeI) in frame with the mKate2 fluorophore. KR2 without the channelrhodopsin N-terminus was amplified from that plasmid and cloned into the pmKate2-N1 plasmid (BamHI/AgeI) in frame with mKate2. Next, mKate2 was replaced by an mKate2 flanked with trafficking sequences^20,21^, resulting in two additional constructs. To further enhance membrane targeting, mKate2 was replaced by eYFP, resulting in a total of six KR2 fluorophore (KR2-FP) fusion constructs with different targeting motifs and fluorophores. An overview of all of the constructs is provided in Fig. 1.

For purification of the recombinant protein, an *E*.*coli* codon-optimized gene encoding for KR2 (UniProt N0DKS8, amino acids 1–280) with a C-terminal TEV protease cleavage site and a HIS_6_ tag (ENLYFQGLEHHHHHH) was synthesized (GenScript, Nanjing, China) and cloned into a pET21a(+) expression vector between the NdeI and XhoI restriction sites.

### Cell culture

ND7/23 cells (Sigma-Aldrich, St. Louis, MO, USA) were cultured in Dulbecco’s Modified Eagle Medium (DMEM) supplemented with 5% (v/v) fetal bovine serum (FBS) and 1 µg/ml penicillin/streptomycin at 37 °C under a 5% CO_2_ atmosphere. For patch-clamp experiments and confocal imaging, 0.5 × 10^5^ cells/ml were seeded on poly-D-lysine coated^41^ glass cover slips in 35-mm Petri dishes. The next day, the cells were transiently transfected using the FuGENE® HD Transfection Reagent (Promega, Madison, WI, USA; 6 µl FuGENE/2 µg DNA per dish) with the respective KR-FP fusion construct. Electrophysiological recordings or confocal imaging were conducted 40–50 h later. For the primary hippocampal cell culture, the hippocampus was dissected from the brains of newborn mice (P0). The dentate gyrus was removed, and the tissue was maintained in Hanks’ Balanced Salt Solution (HBSS). Enzymatic dissociation of the hippocampal neurons was performed at 37 °C for 30 min in a DMEM-based solution containing 4 U/mL papain, 200 µg/mL L-Cysteine, 1 mM CaCl_2_, and 0.5 mM EDTA. The tissue was subsequently incubated for 5 min at 37 °C in inactivating solution containing 2.5 mg/ml albumin and 2.5 mg/ml trypsin inhibitor in DMEM. The inactivating solution was replaced by Neurobasal Medium supplemented with B27 and GlutaMAX. The cells were triturated and plated on an astrocyte feeding layer on glass coverslips coated with poly-D-lysine. The cells were infected with 4.4 × 10^9^ vector genome copies of adeno-associated virus particles at 1 days *in vitro* (DIV) and patch-clamp recordings were obtained between DIV 12–15.

### Confocal imaging

Confocal images were acquired using a FV1000 Olympus (Shinjuku, Tokyo, Japan) confocal laser scanning microscope using a 60× water immersion objective with a numerical aperture of 1.2. The cell membrane was labeled using either octadecyl rhodamine B chloride (R18) for the KR2 variants fused to eYFP, or DiO for variants fused to mKate2. R18 and mKate2 were excited with a 559 nm diode laser (transmissivity 2.5% and 4%, respectively), whereas eYFP and DiO were excited with a 515 nm argon laser (2% transmissivity) and a 488 nm diode laser (1% transmissivity), respectively.

Mean fluorescence intensities (per area) of the respective KR2-FP fusion construct in either the cell membrane (identified by the membrane marker) or within the cell were evaluated (mean background fluorescence was subtracted) for three equatorial slices per cell using a custom Fiji macro and then averaged. Membrane targeting was determined as a factor of mean fluorescence in the cell membrane over the mean fluorescence within the cell, resulting in values above one when the average fluorescence was higher in the membrane compared to the rest of the cell.

### Electrophysiological measurements in *Xenopus laevis* oocytes

Oocyte experiments were conducted as previously described^24^. Briefly, the oocytes were injected with 25–35 ng capped-mRNA per oocyte and incubated at 18 °C in ORI solution supplemented with 5 μM all-*trans* retinal (Sigma-Aldrich, St. Louis, MO, USA). The oocytes were measured 4 days after injection. Two-electrode voltage-clamp (TEVC) was performed using a Turbo TEC-10X amplifier, without transient compensation (npi Electronic, Tamm, Germany). Continuous light was provided by a XBO75/2 75 W Xenon lamp (Carl Zeiss GmbH, Oberkochen, Germany). The light passed through a 400–600 nm broadband filter (Optics Balzers AG, Lichtenstein) with an intensity of approximately 10 mW/mm^2^. The resistances of the microelectrodes were maintained between 0.6 and 1.4 MΩ. Data acquisition and light triggering were controlled using the Axon™ pCLAMP® 9.0 software via the Digidata® 1322A interface (Molecular Devices, Sunnyvale, CA, USA). The currents were recorded at 10 kHz and filtered to 1 kHz using built-in circuits. The photocurrent traces were baseline corrected and filtered to 300 Hz (Gauss-low pass cutoff filter using pCLAMP). The composition of the standard extracellular buffer solution was 100 mM NaCl, 1 mM MgCl_2_, 0.1 mM CaCl_2_, and 5 mM MOPS (pH 7.5). For pH 5 and 10, MOPS was replaced by 5 mM Na-citrate or 5 mM glycine, respectively.

### Patch-clamp recordings in ND7/23 cells

Patch pipettes (2–3.5 MΩ) were prepared from standard wall (inner diameter 0.86 nm, outer diameter 1.50 nm) borosilicate capillaries with filament (Warner Instruments, Hamden, USA) using a horizontal P1000 micropipette puller (Sutter Instrument, Novato, USA). The recordings were performed in the whole-cell configuration on neuronal ND7/23 cells at room temperature^42^. Light was provided by a pE-4000 CoolLED system (CoolLED, Andover, UK) coupled to the optical path of an Axiovert 100 TV inverted microscope (Carl Zeiss, Oberkochen, Germany). If not indicated otherwise, a 525 nm LED (FWHM 31 nm) with a power of 46 mW/mm^2^ in the focal plane of the 40×/1.0 water objective (Carl Zeiss, Oberkochen, Germany) (illuminated area 0.0346 mm^2^) was used for activation. The recorded signals were amplified, filtered at 5 kHz with an ELC-03XS (npi Electronic, Tamm, Germany), and digitized at a sampling rate of 20 kHz using a Digidata® 1440 A digitizer (Molecular Devices, Sunnyvale, USA). The reference electrode was connected to the bath solution via an agar bridge containing 140 mM NaCl. The fluid level (~500 µl) in the bath was kept constant by a Ringer Bath handler MPCU (Lorenz Messgerätebau, Katlenburg-Lindau, Germany) and superfused with at least 5 ml external buffer when the ionic composition on the outside was changed. If not indicated otherwise, standard 110 mM symmetric [Na^+^]_i/e,_ pH_i/e_ 7.2 buffers were used. The ionic composition of the standard buffer and the other buffered solutions is indicated in Supplemental Table S11.

The action spectrum was recorded at a different setup (described here^43^) in which the light was delivered using a Polychrome V monochromator (TILL Photonics, Planegg, Germany), and photon fluxes were adjusted to equal values over the measured spectral range using a motorized neutral density filter wheel (Newport, Irvine, USA). Alternatively, light could be provided at this setup by a tunable laser system (OPOTEK INC., Carlsbad, USA) coupled into the optical path of the microscope to measure photocurrents following a 7-ns laser flash (525 nm) under single turnover conditions. All laser-induced electrical measurements were performed under symmetric buffer conditions to reduce liquid junction potentials (LJP). Data were filtered (low-pass bessel filter) at the amplifier with 100 kHz and digitized with 250 kHz, only small cells (15–20 pF) were selected, and series resistance was compensated up to 90%. The resulting data were binned using a custom MATLAB script to ensure an equal number of data points in each decade of the time domain, and four recordings from the same cell were averaged. For each condition, the recording was repeated for three to five cells, recordings were fitted individually, and then average values were calculated. For presentation purposes, the photocurrents were normalized to the maximal value.

Electrophysiology data were analyzed in Clampfit 10.4, StimFit 0.14^44^ using custom python scripts, Excel 2013, MATLAB, and/or Origin 9.0. Data were corrected for the respective liquid junction potential after recording, which were calculated using the Clampex software; and 0 mV values were linearly interpolated from the neighboring data points after junction potential correction. Apparent off-kinetics were calculated from the biexponential fit, weighting the time constants τ_1_ and τ_2_ according to their respective amplitudes A_1_ and A_2_ using:

$${\tau }_{{\rm{app}}}=(({{\rm{A}}}_{1}\times {{\rm{\tau }}}_{1})+({{\rm{A}}}_{2}\times {{\rm{\tau }}}_{2}))/({{\rm{A}}}_{1}+{{\rm{A}}}_{2}).$$

### Mouse hippocampal neurons electrophysiology

Recordings were performed in the whole-cell current clamp configuration using a MultiClamp 700B axon amplifier at room temperature. The signals were digitized with a Digidata® 1550A digitizer (Molecular Devices) and recorded using the pCLAMP® 9 software. Data were acquired at 50 kHz and filtered at 10 kHz. Electrodes with 2–3 MΩ resistance were pulled (Sutter Instrument, Novato, USA) from borosilicate glass (Harvard Apparatus; PG150T-15). Compensation for series resistance and capacitance neutralization was achieved using the bridge-balance and the MultiClamp 700B capacitance neutralization functions. Standard intracellular solutions contained: 17.8 mM HEPES, 135 mM KGluc, 4.6 mM MgCl_2_, 4 mM MgATP, 0.3 mM NaGTP, 1 mM EGTA, 12 mM Na_2_Phosphocreatine, and 50 mM phosphocreatine kinase, pH 7.3. Standard extracellular solutions contained: 10 mM HEPES, 140 mM NaCl, 2.4 mM KCl, 2 mM CaCl_2_, 4 mM MgCl_2_, and 10 mM glucose, pH 7.4. The extracellular buffer also contained the synaptic transmission blockers NBQX (10 µM), Picrotoxin (100 µM), and CCPene (10 µM). The respective liquid junction potential was calculated to be 17.2 mV using Clampex software and corrected after recording, if indicated.

A 540 nm Mightex Collimated LED light source (LCS-0540-14) was used for all experiments, whereas the shape and intensity of the stimulus was delivered using the SLC-AA04-US controller coupled to a Digidata digitizer. The Mightex light source was coupled to an Olympus IX-73 microscope, and light was delivered through a LUCPLFLN 60×/0.7 Olympus objective. The illumination field was 0.102 mm^2^. Electrophysiological data were analyzed using Clampfit 10, Origin 9, and GraphPad Prism 5. Action potential numbers were calculated using the event detection function in Clampfit. The smallest AP threshold was set for the complete ramp, and this threshold was calculated for each individual neuron. Two group comparison statistics were conducted using the Wilcoxon Rank Sum Test. Three group comparison statistics was made using the Kruskal Wallis test followed by the Dunn’s post-hoc test.

### Purification of recombinant KR2

Protein transformation, expression, and purification was performed as previously reported^15^. The expression plasmid (pet21a+) was transformed into C41(DE3) *E*. *coli* cells. Protein expression was induced with 0.5 mM isopropyl β-D-thiogalactopyranoside (IPTG; Carl Roth GmbH, Karlsruhe, Germany) and the LB culture medium was supplemented with 5 µM all-*trans* retinal (ATR; Sigma-Aldrich, St. Louis, USA). The cells were harvested 3 hours after induction and disrupted using an EmulsiFlex-C3 Homogenizer (AVESTIN Inc., Ottawa, Canada). The membrane fraction was collected by ultracentrifugation (45,000 rpm) for 1 h at 4 °C (Type 45 Ti; Beckman Inc., Indianapolis, USA). The membrane pellet was resuspended in buffer containing 50 mM Tris-HCl (pH 8.0), 300 mM NaCl, 0.1 mM PMSF, 1.5% n-dodecyl-β-D-maltoside (DDM, GLYCON Biochemicals GmbH, Luckenwalde, Germany), and 0.3% cholesteryl hemisuccinate (CHS, Sigma-Aldrich, St. Louis, USA) and stirred overnight for solubilization. The insoluble fraction was removed by ultracentrifugation (200,000 × *g*, 1 h at 4 °C). The KR2 protein was purified by Ni-NTA affinity and size-exclusion chromatography using an ÄKTAxpress protein purification system (GE Healthcare Life Science, Chicago, USA) configured with a HisTrap HP Ni-NTA column and a 125 mL Superdex 200 PG column. The protein fractions collected were concentrated in buffer containing 50 mM Tris-HCl (pH 8.0), 150 mM NaCl, 0.1 mM PMSF, 0.02% DDM, and 0.004% CHS.

To perform measurements in varied [H^+^] and [Na^+^], rapid buffer exchange was performed using PD-10 Desalting Columns with Sephadex G-25 resin (GE Healthcare Life Science, Chicago, USA). The high/low [Na^+^] in neutral pH was achieved using buffer consisting of 110 or 1 mM NaCl and 50 mM Tris-HCl (pH 7.2) respectively. The low [Na^+^] and low [H^+^] was achieved using buffer consisting of 1 mM NaCl and 50 mM glycine-NMG (pH 9.0). The high [K^+^] was achieved using buffer consisting of 110 mM KCl, 1 mM NaCl, and 50 mM Tris-HCl (pH 7.2). To determine the effect of pH in high [Na^+^] the buffer consisted of 150 mM NaCl and 50 mM HEPES-KOH (pH 6.4), or 50 mM Tris-HCl (pH 8.0), or 50 mM Glycerin-KOH (pH 9.0).

### UV-visible spectroscopy

The UV-Vis spectroscopic measurements were performed as previously reported^45^ at 22 °C. Dark state absorption spectra were recorded using a Cary 300 spectrophotometer (Varian Inc., Palo Alto, USA) at a spectral resolution of 1.6 nm. A LKS.60 modified flash photolysis system (Applied Photohysics Ltd., Leatherhead, UK) was used to measure microsecond-to-second changes in transient absorbance in multi-wavelength datasets (resolution of 0.4 nm). To excite the sample, the laser pulse was tuned to 530 nm using a tunable optical parametric oscillator (MagicPrism, Opotek Inc., Carlsbad, CA, USA), which was pumped via the third harmonic of a Nd:YAG laser (BrilliantB, Quantel, Les Ulis, France). The laser energy was adjusted to 5 mJ/shot and a pulse duration of 10 ns. A 150 W Xenon lamp (Osram, München, Germany) was used to monitor the changes in the absorption. The transient spectra were recorded using an Andor iStar ICCD camera (DH734; Andor Technology Ltd, Belfast, Ireland) at 181 different time points between 1 µs and 1 s (30 points per decade, isologarithmically). The transient spectra of the samples in buffer containing 150 mM NaCl were recorded at 46 different time points between 10 ns and 10 s (5 data points per decade, isologarithmically), whereas the spectra of the samples in buffer containing 110 mM KCl were recorded at 46 different time points between 1 µs and 1 s. To ensure complete recovery of the dark state, the sample was held in the dark for 25 s before the following recording. The resulting data sets were averaged over at least 10 cycles.

The primary data analysis was performed using MATLAB R2016b (The MathWorks, Natick, MA) to calculate difference spectra and reconstruct the three-dimensional spectra. Global analysis of the spectral datasets was performed with Glotaran 1.5.1 (Vrije Universiteit Amsterdam, Netherlands)^46,47^. Singular value decomposition (SVD) analysis of the data was used to determine the number of significant components needed to reconstruct the signal from the data sets, enabling noise reduction. The τ values and the intermediate spectra (EADS and DADS) were extracted from the data sets using global analysis. The sequential model explored the spectral evolution and produced the Evolution Associated Difference Spectra (EADS), representing the species-associated difference spectra. The parallel scheme explored the loss and gain of absorption with a certain lifetime and produced the Decay Associated Difference Spectra (DADS), representing the decay and rise of the states. Both schemes are mathematically equivalent and result in exactly the same τ values^48^. For additional analysis Excel 2016 and Origin 9.0 were used.

### Statistics

If not indicated otherwise, data are represented as mean ± standard deviation (SD). Statistical analyses were performed in R (version 3.3.2) using the Wilcoxon Rank Sum Test. Significance levels are indicated as: *=0.01 < p < 0.05, **=0.001 < p < 0.01, ***=p < 0.001, and ns = not significant.

## Electronic supplementary material

Supplemental figures 1-10, Supplemental table 11
