# Supplementary material for: Electrical properties, substrate specificity and optogenetic potential of the engineered light-driven sodium pump eKR2
Source: Sci Rep. 2018 Jun 18;8:9316. doi: 10.1038/s41598-018-27690-w (PMC6006383; doi:10.1038/s41598-018-27690-w)
Supplement: Supplementary file 1 — Supplemental figures 1-10, Supplemental table 11 [file 41598_2018_27690_MOESM1_ESM.pdf]

Supplementary Information for:

Electrical properties, substrate specificity and optogenetic potential of the engineered light-driven sodium pump eKR2

Christiane Grimm[1*], Arita Silapetere[1], Arend Vogt[1], Yinth Andrea Bernal Sierra[1], Peter Hegemann[1*]

[1]Experimental Biophysics, Institute for Biology, Humboldt-Universität zu Berlin, Invalidenstr. 42, 10115 Berlin, Germany

*Corresponding Authors:

Christiane Grimm
Address: Invalidenstrasse 42, 10115 Berlin, Germany
Phone: +49-30-2093-8697
E-mail: grimmchristiane@outlook.com

Peter Hegemann
Address: Invalidenstrasse 42, 10115 Berlin, Germany
Phone: +49-30-2093-8830
E-mail: hegemann@rz.hu-berlin.de

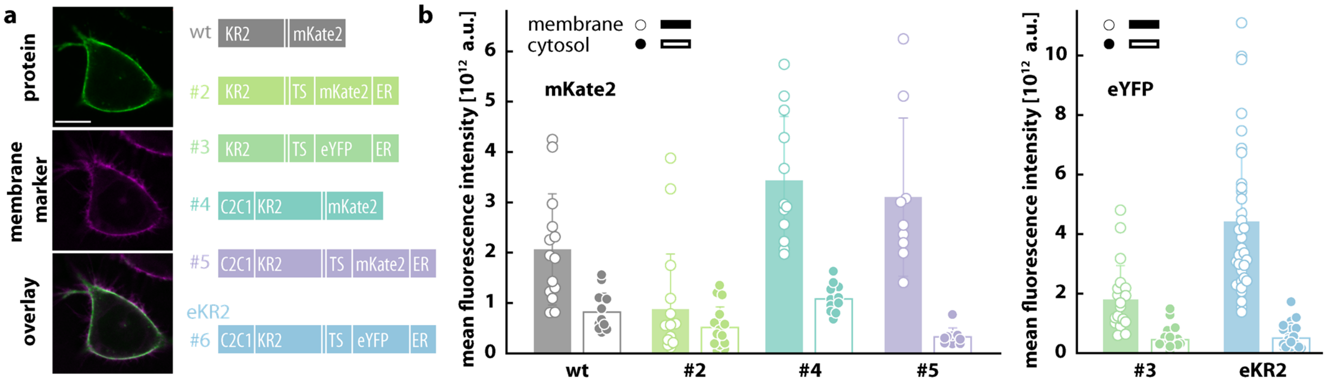

Supplemental figure S1 Membrane localization of targeting constructs . a) Representative confocal images of eKR2 (equatorial 0.5 µm slice) with the fluorescene of the protein in green (top), the membrane marker in magenta (middle) and their co-localization in white (bottom), which were used to determine the membrane localization for all constructs **b)** Average fluorescence (±SD) of all constructs in the membrane (open circles) and the cytosol (filled circles). Left: mKate2-tagged constructs; right: eYFP tagged constructs.

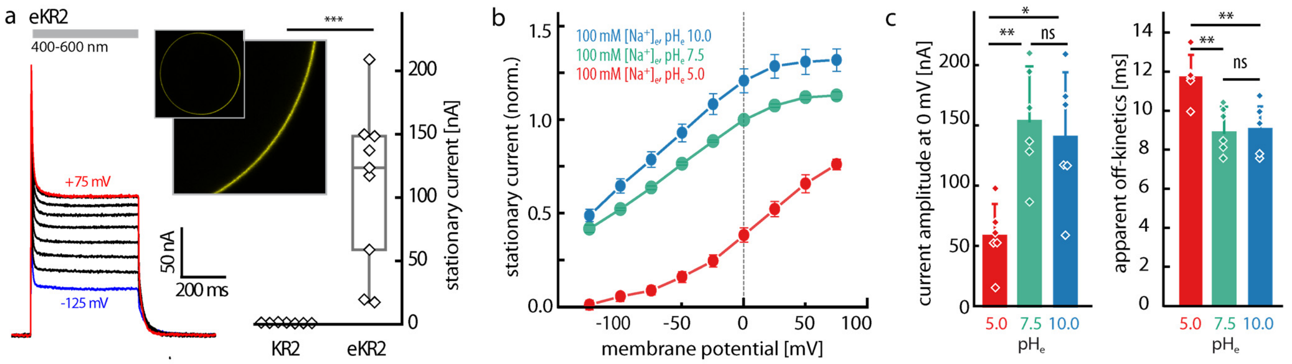

Supplemental figure S2 Electrophysiological measurements from *Xenopus* oocytes. a) Representative photocurrents of eKR2 at different holding potentials (25 mV steps, pH_e 7.5, 100 mM external $[Na^+]_e$) with comparison of I_s from wt KR2 (no fluorophore) and eKR2 **(**eYFP**)** at 0 mV (100 mM $[Na^+]_e$, pH_e 7.5) and confocal images of eKR2 (10 µm equatorial slice) with eYFP fluorescence in yellow. **b)** I_s of eKR2 at holding potentials between -125 and +75 mV at varying extracellular pH_e; displayed as mean ± SEM, n=7, 9, 5. **c)** I_s and apparent off-kinetics at varying extracellular pH and high $[Na^+]_e$ (conditions from b); two-sample t-test * - $0.01 < p < 0.05$, ** - $0.001 < p < 0.01$, *** - $p < 0.001$, ns - not significant.

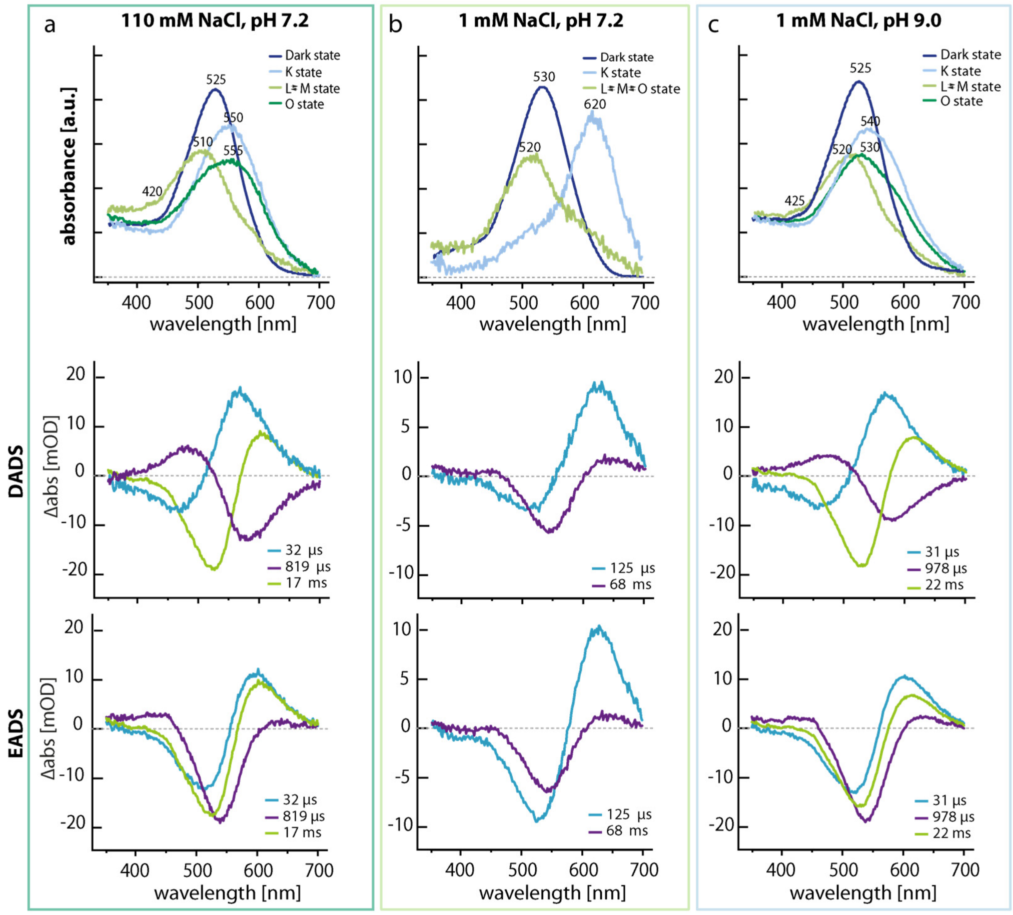

Supplemental figure S3 UV-visible spectroscopy of recombinant KR2. Measured dark state and determined photointermediate absorption spectra (top row). The globally analysed flash photolysis data using parallel (middle) or sequential (bottom) kinetic scheme with the resulting photointermediate decay τ values indicated in the right bottom corner of the panels. The spectra are compared between different pH and ionic conditions **a)** 110 mM NaCl, pH 7.2, **b)** 1 mM NaCl, pH 7.2, **c)** 1 mM NaCl, pH 9.0. Decay associated difference spectra (DADS) show the loss and gain of the absorption, while the evolution associated difference spectra (EADS) show the spectral evolution.

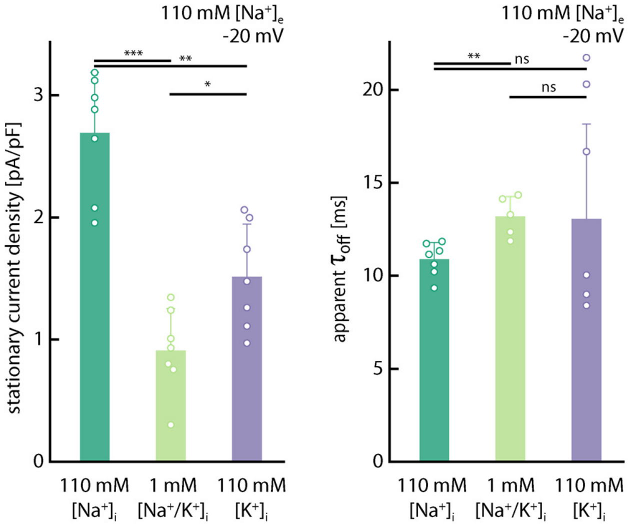

Supplemental figure S4 Variation of intracellular $[K^+]_i$. Stationary photocurrents of eKR2 upon excitation with a 525 nm LED (46 mW mm^{-2}) at -20 mV holding potential (110 mM $[Na]_e$ and pH_e 7.2) and varying intracellular $[K^+]_i$ (left) together with the corresponding apparent kinetics of the current decline after light-off (right); displayed as mean ± SD with individual data points; significance tested using Wilcoxon Rank Sum test * - $0.01 < p < 0.05$, ** - $0.001 < p < 0.01$, *** - $p < 0.001$, ns - not significant.

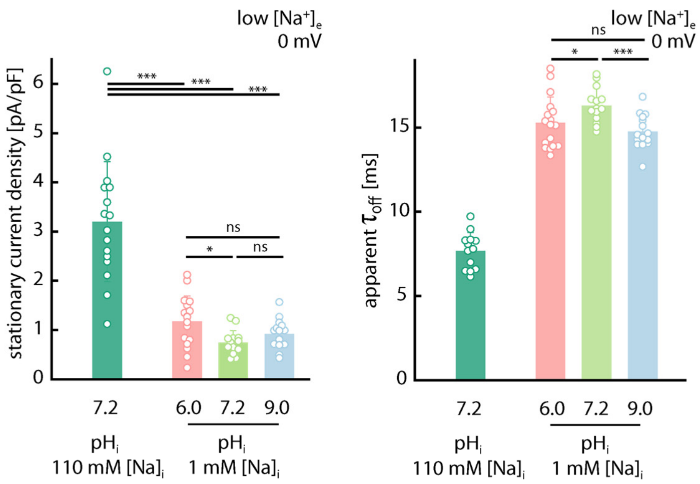

Supplemental figure S5 Variation of intracellular [Na]$_i$ and pH$_i$ at low extracellular [Na$^+$]$_e$. Stationary photocurrents of eKR2 at 1 mM [Na$^+$]$_e$, pH$_e$ 7.2 (0 mV holding potential) and varying intracellular [Na$^+$]$_i$ and pH$_i$ (left) together with the corresponding apparent kinetics of the current decline after light-off (right); displayed as mean ± SD with individual data points; significance tested using Wilcoxon Rank Sum test * - 0.01 < p < 0.05, ** - 0.001 < p < 0.01, *** - p < 0.001, ns - not significant.

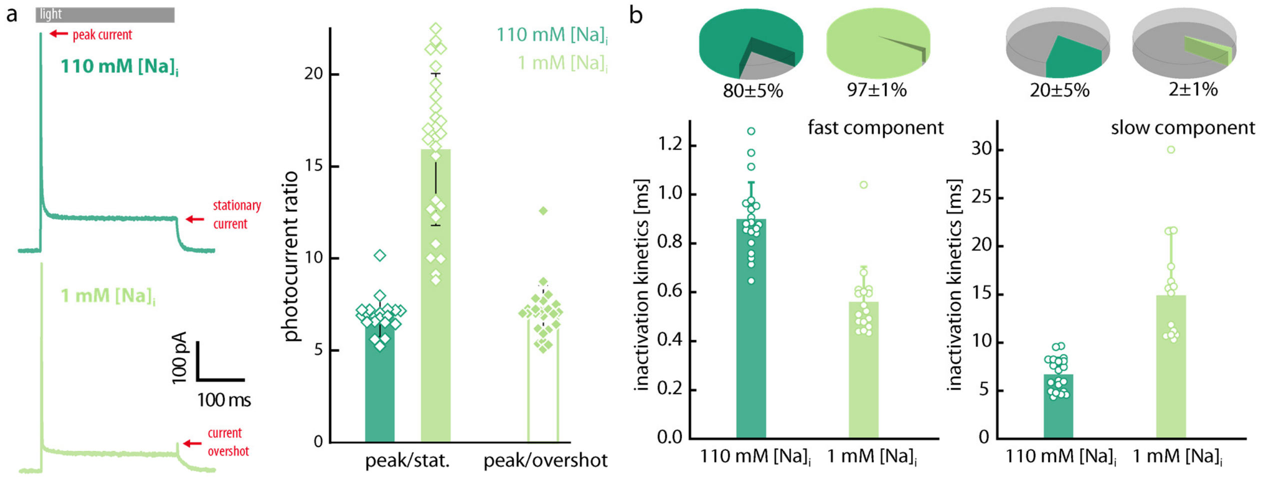

Supplemental figure S6 Inactivation from I_P to I_S at high and low internal sodium (110 mM $[Na^+]_e$, pH_e 7.2). a) Ratio of peak to stationary current at high and low intracellular $[Na^+]_i$ and ratio from peak current to the current overshot after light-off only visible at low $[Na^+]_i$. **b)** Inactivation kinetics from I_P to I_S (0 mV) was fitted biexponetially and revealed a sub-ms fast component (left), which accelerates and increases in relative amplitude (pie chart) when internal $[Na^+]_i$ is reduced, and a slow component (right), which decelerates and decreases in amplitude when the internal $[Na^+]_i$ is lowered; displayed as mean±SD with individual data points.

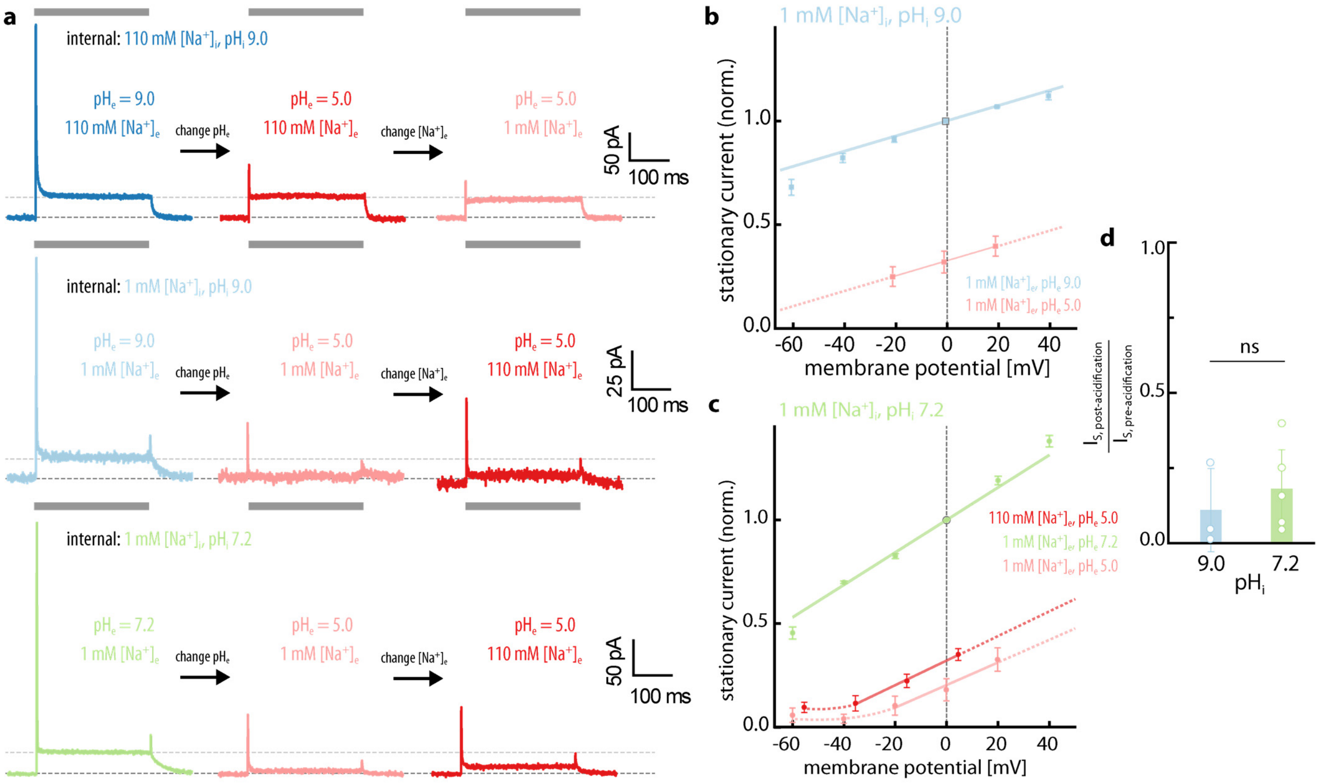

Supplemental figure S7 Influence of acidic pH_e on eKR2 photocurrents at different pH and Na^+-gradients in ND7/23 cells. a) Representative photocurrent traces at 0 mV; traces in one row from same cell. From symmetric conditions (left column) the external pH is lowered (middle column) and then a Na^+-gradient is established (right column) Top: high $[Na^+]_i$, pH_i 9.0, middle: low $[Na^+]_i$, pH_i 9.0 and bottom: low $[Na^+]_i$, pH_i 7.2 **b)** Current-voltage relations corresponding to middle row (left and middle column) in panel a) at low $[Na^+]_i$, pH_i 9.0 and pH_e 5.0 (1 mM $[Na^+]_e$); LJP corrected, normalized to 0 mV symmetric condition, mean ± SEM, n = 2 and 2 **c)** Current-voltage relations at external pH 5.0 (high and low $[Na^+]_e$) at low $[Na^+]_i$, pH_i 7.2 corresponding to panel a) bottom row; LJP corrected, normalized to 0 mV symmetric condtion, mean ± SEM, n = 6, 6 and 6 **d)** Reduction of I_s upon extracellular acidification (pH_e 5.0) from symmetric pH at 0 mV ($pH_{i/e}$ 9.0 or 7.2 and low $[Na^+]_{i/e}$). Corresponds to panel a) middle and bottom row with ratio of I_s from middle and left column; mean ± SD and individual data points.

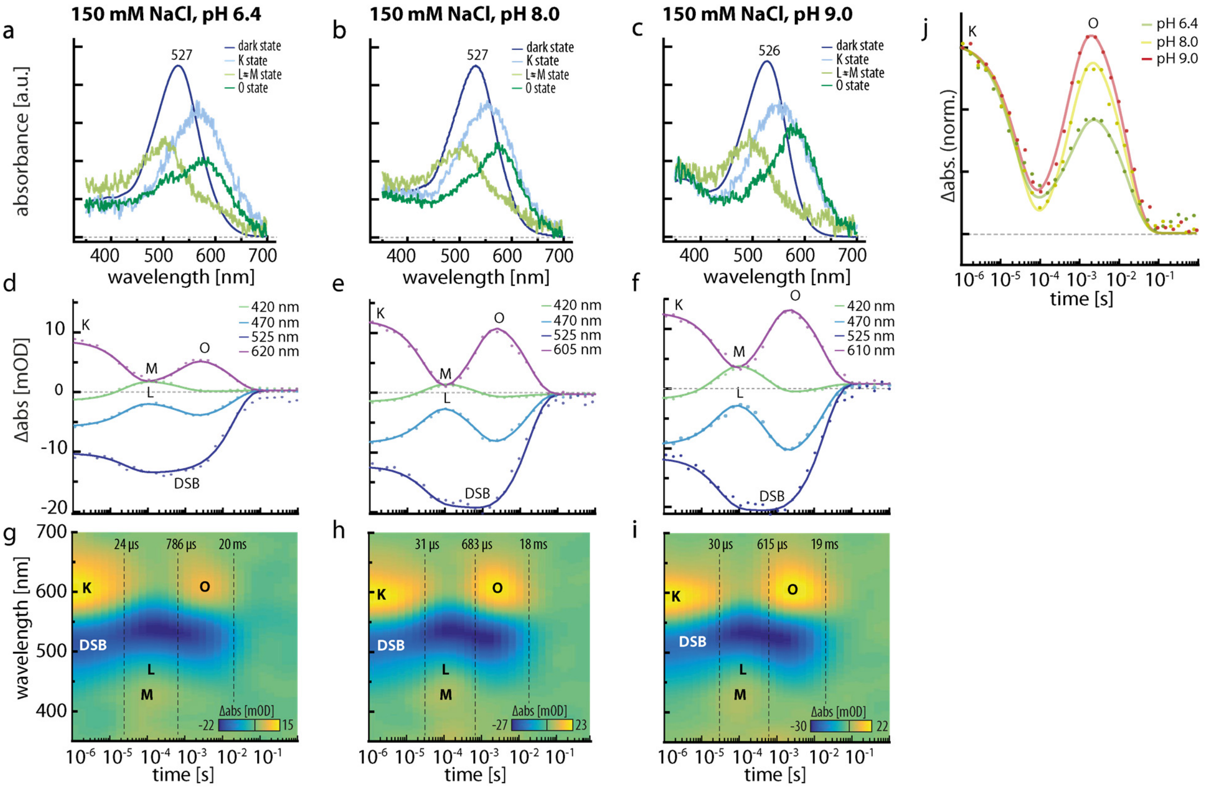

Supplemental figure S8 Absorption spectra, time traces and reconstructed absorption difference surface plots of recombinant KR2 at 150 mM NaCl and varied pH. Spectral data of purified KR2 recorded in buffer with 150 mM NaCl and increasing pH value – 6.4 (left), 8.0 (middle), 9.0 (right). **a-c)** Dark state absorbance and calculated absorption spectra of photointermediates **d-f**) Time traces of absorption differences from 1 µs to 1 s at 420 nm (green), 470 nm (light blue), 525 nm (dark blue), 620 nm (magenta). Time traces extracted from the global fit are represented with solid lines and the time traces extracted from SVD filtered data are shown with point diagrams. Wavelength is chosen according to the maximal absorption difference and slightly varies between the conditions. **g-i**) Reconstructed absorption difference surface plot and the consequent photointermediate decay τ values originating from global analysis. **j)** Absorption difference of the O-state at varying pH values normalized to respective K-state absorption difference.

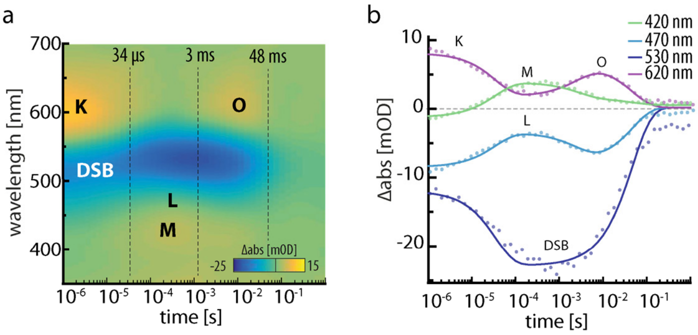

Supplemental figure S9 Transient absorption spectra of recombinant KR2 in high K+. a) Reconstructed absorption difference surface plot and the consequent photointermediate decay τ values originating from global analysis of purified KR2 in 110 mM K+-buffer. **b**) Time traces from 1 µs to 1 s at 420 nm (green), 470 nm (light blue), 525 nm (dark blue), 620 nm (magenta). Time traces extracted from the global fit are represented with solid lines and the time traces extracted from SVD filtered data are shown with point diagrams.

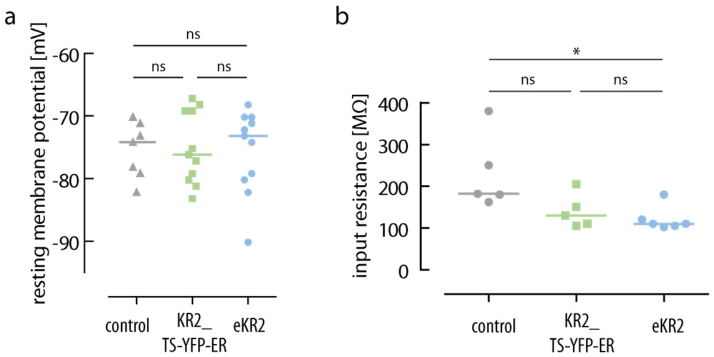

Supplemental figure S10 Electrophysiological properties of mouse hippocampal neurons in culture after infection with AAV2/9 viruses. **a)** Resting membrane potential values after junction potential correction. **b)** Input resistance in whole cell mode. For all data a Kruskal-Wallis test was made followed by the multicomparison Dunn's post-hoc test.

Supplemental table S11 Electrophysiology buffer. Ionic compositions of extra- and intracellular buffered solutions for recordings from ND7/23 cells; pH values were adjusted to the indicated values using 1 M NMG and 100 mM citric acid, while glucose was added to reach 290 mOsm for intra and 320 mOsm for extracellular solutions. All concentrations given in mM.

intracellular	110 Na^+, pH 7.2	1 Na^+, pH 7.2	0.1 Na^+, pH 7.2	110 Na^+, pH 9.0	1 Na^+, pH 9.0	1 Na^+, pH 6
**KCl**	1	1	1	1	1	1
**NaCl**	110	1	0.1	110	1	1
**$MgCl_2$**	2	2	2	2	2	2
**$CaCl_2$**	2	2	2	2	2	2
**CsCl**	1	1	1	1	1	1
**HCl**	-	110	110	110	110	110
**EGTA**	10	10	10	10	10	10
**HEPES**	10	10	10	0	0	0
**TRIS**	0	0	0	10	10	0
**MES**	0	0	0	0	0	10

extracellular	110 Na^+, pH 7.2	1 Na^+, pH 7.2	110 Na^+, pH 9.0	1 Na^+, pH 9.0	110 Na^+, pH 5.0	1 Na^+, pH 5.0
**KCl**	1	1	1	1	1	1
**NaCl**	110	1	110	1	110	1
**$MgCl_2$**	2	2	2	2	2	2
**$CaCl_2$**	2	2	2	2	2	2
**CsCl**	1	1	1	1	1	1
**HCl**	0	110	0	110	0	110
**HEPES**	10	10	0	0	0	0
**TRIS**	0	0	10	10	0	0
**Citric acid**	0	0	0	0	5	5
